# Characterization of the Biological Activity of the Ethanolic Extract from the Roots of *Cannabis sativa* L. Grown in Aeroponics

**DOI:** 10.3390/antiox11050860

**Published:** 2022-04-27

**Authors:** Fabio Ferrini, Sabrina Donati Zeppa, Daniele Fraternale, Vittoria Carrabs, Giosuè Annibalini, Giancarlo Verardo, Andrea Gorassini, Maria Cristina Albertini, Tariq Ismail, Carmela Fimognari, Piero Sestili

**Affiliations:** 1 Department of Biomolecular Sciences, University of Urbino Carlo Bo, Via Saffi, 2, 61029 Urbino, Italy; sabrina.zeppa@uniurb.it (S.D.Z.); daniele.fraternale@uniurb.it (D.F.); v.carrabs@campus.uniurb.it (V.C.); giosue.annibalini@uniurb.it (G.A.); maria.albertini@uniurb.it (M.C.A.); piero.sestili@uniurb.it (P.S.); 2 Department of Agricultural, Food, Environmental and Animal Sciences, University of Udine, 33100 Udine, Italy; giancarlo.verardo@uniud.it; 3 Department of Humanities and Cultural Heritage, University of Udine, 33100 Udine, Italy; andrea.gorassini@uniud.it; 4 Institute of Food Science and Nutrition, Bahauddin Zakariya University, Multan 60800, Pakistan; tariqismail@bzu.edu.pk; 5 Department of Food Technology, Engineering and Nutrition, Lund University, SE-221 00 Lund, Sweden; 6 Dipartimento di Scienze per la Qualita della Vita, Alma Mater Studiorum—Universita di Bologna, 47921 Rimini, Italy; carmela.fimognari@unibo.it

**Keywords:** *Cannabis sativa* L., aeroponic roots, β-sitosterol, epi-friedelanol, friedelin, antioxidant, anti-inflammatory

## Abstract

*Cannabis sativa* var. Kompolti, a variety routinely used for food production purposes, is characterized by a low concentration of psychoactive molecules, although containing many other biologically attractive metabolites in all parts of the plant, including the roots. In the present work, we evaluate the specific biological activities of the roots’ extract from plants cultivated through aeroponics, an affordable and reliable method facilitating the isolation and processing of roots, with the advantage of being suitable for industrial scale-up. Furthermore, aeroponics results in an increased net accumulation of the most biologically attractive constituents (β-sitosterol, friedelin and epi-friedelanol) found in the roots. The ethanolic extract of the aeroponic roots of *C. sativa* (APEX) and its separate components are studied to evaluate their anti-inflammatory (modulation of the expression level of specific markers upon LPS stimulation in U937 cells, such as *IL-6*, *IL-8*, *TNF*-α, *IkB*-α, *iNOS*, *IRAK-1* and *miR-146a*) and antioxidant (in either acellular or cellular settings) activities. The APEX anti-inflammatory and antioxidant capacities are also functionally benchmarked using the wound-healing assay. On the whole, the data obtained show that APEX and its main components showed significant anti-inflammatory and antioxidant activities, which may render the exploitation of roots as a source of natural antioxidants and anti-inflammatory agents highly attractive, with the additional technical and economic advantages of aeroponics compared to soil cultivation.

## 1. Introduction

*Cannabis* L. (Linneaus, 1753) is a genus of angiosperms belonging to the Cannabaceae family. According to the prevailing guidelines, hemp includes a single species, called *Cannabis sativa*, which is the most historically widespread plant in the West [1].

The variability of secondary metabolites in the plant determines the taxonomy in two subgroups or chemotypes, depending on the enzyme involved in cannabinoid biosynthesis [2]. The CBD (cannabidiol) chemotype, characterized by the prevalence of the enzyme CBDA-synthase, is cultivated for agro-industrial and therapeutic uses, while the THC (tetrahydrocannabinol) chemotype, characterized by the enzyme THCA-synthase, is used in the pharmaceutical industry as a source of THC, but also for recreational purposes due to the powerful and well-known psychotropic activity [3].

Obviously, despite the biological and pharmacological properties of *C. sativa* being known to popular medicine since time immemorial, the pharmacological exploitation of this plant has been severely limited due to the risk of abuse due to the presence of THC. For this reason, the agricultural–industrial sector has resumed investing in the cultivation and exploitation of the so-called “light hemp”, i.e., the varieties of *C. sativa* with low THC content (not exceeding 0.2%), for pharmaceutical, nutraceutical and cosmeceutical purposes. Furthermore, these *C. sativa* subspecies contain numerous bioactive molecules different from CBD and THC in all of the plant’s parts [4], and the agricultural industry is interested in developing cultivation methods that also increase the yield of the secondary metabolites present in the plant parts [5].

Today, the subspecies *sativa* is widely and legally cultivated in many Western countries for the production of CBDs—the most important non-psychoactive cannabinoids—as well as of seeds, which are an excellent source of lipids, proteins, carbohydrates, minerals, vitamins, amino acids, essential fatty acids and insoluble fiber [6].

Recently, the secondary metabolites in each part of the *C. sativa* plant have been characterized, highlighting the presence of the triterpenoids friedelin (FR) and epi-friedelanol (EFR) and of the sterols β-sitosterol (ST), stigmasterol and campesterol in the roots [2] where neither THC nor CBD are present.

In the present work, *C. sativa* subsp. *sativa* var. Kompolti—a light, legal variety routinely used in the food industry—was cultivated through aeroponic culture [7], in which the plants grow free of contaminants in a highly controlled manner, leading to an overgrowth of roots in comparison to traditional cultivation in soil [6]. The aeroponics culture also provides a greater yield of *C. sativa* roots’ secondary bioactive metabolites, FR, EFR and ST. This sterol exhibits antihypercholesterolemic and antitumoral properties [8,9,10]. Furthermore, ST has been shown to inhibit aromatase and 5-alpha-reductase, an activity exploited to treat pathologies such as benign prostatic hyperplasia and androgenetic alopecia [11,12,13,14,15,16].

FR, a pentacyclic triterpenoid contained in a variety of plants, has been shown to display a wide spectrum of anti-inflammatory, antioxidant, antipyretic, anticarcinogenic and antitumor effects [17,18,19,20,21,22,23,24,25,26].

Another molecule contained in fairly high amounts in *C. Sativa* roots as well as in other plants’ roots [27,28,29,30,31] is EFR, a pentacyclic triterpenoid differing from FR for the presence of cyclohexanol in place of cyclohexanone. EFR reportedly possesses anticancer [32,33,34], anti-inflammatory [33] and antisenescence activities [35].

With the aim of exploiting *C. sativa* aeroponic root preparations for health-promoting purposes, the present study was undertaken to quantify and mechanistically analyze the anti-inflammatory and antioxidant activities—both inferable on the basis of the above ethnopharmacological reports—of the whole ethanolic extract of the aeroponic roots of *C. sativa* (APEX) and, for comparative purposes, of its main components.

## 2. Materials and Methods

### 2.1. Chemicals and Reagents

Analytical grade extraction solvents, cholesterol, β-sitosterol, stigmasterol, campesterol, friedelin and saturated *n*-alkanes standard (C7–C40) were purchased from Sigma-Aldrich (Milan, Italy).

### 2.2. Plant Material and Cultures

*C. sativa* Kompolti seeds were obtained from Appennino Farm, Gaggio Montano, Bologna (Italy), lot B30756201900001, and were germinated in filter paper humidified with distilled water—10 seeds in a 14 cm diameter glass Petri dish—in the dark, at a constant temperature of 25 °C. Four days later, the rooted seeds were transferred into 5.5 cm diameter/6.0 cm height plastic pots filled with a mixture of 50% peat and 50% vermiculite wetted with Hoagland’s half-strength nutrient solution up to the transplanting stage. The pots were maintained in a climatic cell with a photoperiod of 18 h until the development of the first two true leaves. At this point, five *C. sativa* seedlings of uniform size were selected. The base of the stem of each seedling was placed in a reticulated pot for aeroponics, and mounted in a tub for aeroponic cultivation (50.0 cm × 50.0 cm × 34.0 cm in height, hosting five plants each time). Complete Hoagland’s nutrient solution was sprayed at the roots of the plants for a duration of 15 min per hour. The electrical conductivity (EC) and pH of the nutrient solution were routinely checked. The aeroponics culture system was irradiated with high-pressure sodium lamps (Sonlight AGRO 250W grow + bloom). The photosynthetic photon flux density was about 150 μmol/m^2^/s. Temperatures during the light and dark periods were maintained at 27 ± 1 °C and 22 ± 1 °C, respectively, with a relative humidity of 65 ± 5% and CO_2_ concentration of 670 ± 30 μmol/mol.

### 2.3. Gas Chromatography (GC-MS, GC-FID)

GC-MS analyses were performed using a Trace GC Ultra gas chromatograph coupled to an ion-trap mass spectrometer (ITMS) detector Polaris Q and equipped with a split–splitless injector (Thermo Fisher Scientific, Milan, Italy). In addition, 30 m × 0.25 mm columns with a 0.1 µm film thickness and a fused silica SLB-5ms were used (Supelco, Sigma-Aldrich, Milan, Italy). The initial oven temperature was a 240 °C–280 °C ramp with a 2 °C/min increase and maintained at 280 °C for 5 min; the temperature was then raised to 310 °C at a rate of 10 °C/min and kept at maximum temperature for 7 min. Samples (1 μL) were injected into the split (1:10) mode. The injector, transfer line and ion source were set at 280, 280 and 200 °C, respectively. Helium was used as the carrier gas. The mass spectra were recorded in electron ionization (EI) mode at 70 eV, a mass range from *m/z* 50 to 650, at a 0.8 scan/s scan rate.

A Fisons GC 8000 series gas chromatograph (Fisons Instruments, Milan, Italy) was used for the quantitation of secondary metabolites. The separation was carried out with a 30 m × 0.250 mm × 0.25 μm film thickness capillary column DB-5MS UI (Agilent, J&W, Mila, Italy). The initial oven temperature was 240 °C ramped to 280 °C at 2 °C/min, kept at 280 °C for 10 min and then increased by 10 °C/min to 310 °C, and maintained at this temperature for 15 min. The detectors were set at 280 °C. Hydrogen was used as the carrier gas. Peak areas were integrated with a Varian Galaxie Workstation (Agilent Technologies, Cernusco sul Naviglio (MI), Italy).

### 2.4. Ethanolic Extract of Aeroponic Plant Roots (APEX)

The *C. sativa* roots were collected after eight weeks of cultivation in the aeroponic system. Between 2 and 5 g of roots were chopped with scissors and subsequently ground by pestle and mortar, and finally, 100 mL of ethanol/water (80:20) was added. The chemical characterization of this extract by GC-MS has been previously described by Ferrini et al., 2021 [6].

Everything was placed in an Erlenmeyer flask and subjected to a 24 h magnetic agitation. At the end, the final product was aliquoted in centrifuge tubes and dried using a Savant concentrator (Thermo Scientific, San Jose, CA, USA).

The samples were cold-spun to avoid the degradation of the thermolabile compounds contained in the *C. sativa* roots. After about 6 h, the dry extract was weighed: a total of 30 ± 1.05 mg of dry extract was obtained from the original 5 g of roots.

The dried samples were stored at 4 °C until use. For the experiments, the dried APEX was resuspended in DMSO.

### 2.5. Cell Culture

Human promonocytic U937 cells (Istituto Zooprofilattico Biobank, Brescia, Italy) were cultured in suspension in RPMI 1640 medium (Corning, New York, NY, USA) supplemented with antibiotics (100 U/mL penicillin, 100 μg/mL streptomycin), 1.2 mM glutamine and 10% fetal bovine serum, at 37 °C in a humidified atmosphere of 5% CO_2_.

The human articular chondrocyte cell line HC-a (Sciencell, Carlsbad, CA, USA) was maintained in DMEM (Corning, New York, NY, USA) supplemented with antibiotics (100 U/mL penicillin, 100 μg/mL streptomycin), 1.2 mM glutamine and 10% fetal bovine serum, at 37 °C in a humidified atmosphere of 5% CO_2_.

HC-a cultures were passaged on, reaching 80% confluence, using a trypsin–EDTA solution (Sigma-Aldrich, Milan, Italy). The experiments were performed in the third culture passage (P3).

### 2.6. DPPH Radical Scavenging Activity

The DPPH free-radical scavenging assay was performed as reported in Sestili et al. [36]. The decrease in absorbance (517 nm) was recorded after 10 min at room temperature in the dark.

The scavenger effect was calculated as % = ((A517 nm of blank − A517 nm of samples)/A517 nm of blank) × 100.

The EC_50_ values were calculated by linear regression from the dose/response plots and represent the concentration required to provide 50% free-radical scavenging activity.

### 2.7. Chelating Effect on Fe^2+^

The chelating effect on Fe^2+^ was measured as previously reported by Saltarelli et al. [37]. 200 μL of APEX, FR, ST and EFR were mixed with 740 μL of deionized water; the mixture was reacted with 20 μL FeSO_4_ (2 mM) and 40 μL ferrozine (5 mM) for 10 min and then the absorbance at 562 nm determined.

The chelating activity was calculated as % = ((A562 nm of blank − A562 nm of sample)/A562 nm of blank) × 100.

The EC_50_ values represent the concentrations at which ferrous ions are chelated by 50%.

### 2.8. Cell Treatments

In order to evaluate the antioxidant activity, stock solutions of H_2_O_2_ were freshly prepared in distilled water and diluted to the proper concentrations. APEX, FR, ST and EFR were added to cultures 24 h before the addition of the oxidant; oxidative treatments were performed in 1 mL of medium in six-well TC plate, containing 4 × 10^5^ cells/treatment condition by adding 100 µM H_2_O_2_ for 1 h. Cells were washed with 2 mL of saline A (8.182 g l−1NaCl, 0.372 g l−1KCl, 0.336 g l−1NaHCO_3_ and 0.9 g l−1glucose) and analyzed either immediately or following a further 24 or 48 h post-challenge growth in fresh, drug-free medium. 

To evaluate the anti-inflammatory properties of APEX, FR, ST and EFR, U937 cells (1 × 10^6^ cells/mL) were stimulated for 12 h with 1 µg/mL of lipopolysaccharide (LPS; Sigma -Aldrich, St. Louis, MO, USA). APEX, FR, ST and EFR were added 24 h before LPS stimulation.

At the end of the 12 h LPS stimulus, the cells were assayed for inflammatory marker gene expression analysis. In other experiments, after exposure to LPS, the cells were resuspended in fresh medium and grown for a further 24 h. At the end of this stage, the medium was collected to quantify the IL-6 protein level with an ELISA assay.

### 2.9. Cell Viability Assay

Cell number and viability were determined using the trypan blue exclusion assay. Briefly, an aliquot of cell suspension was diluted 1:1 with 0.4% trypan blue and the cells were counted with a LUNA-II™ Automated Cell Counter (Logos biosystem, Dongan-gu Anyang-si, South Korea). The results are expressed as the percent ratio between the number of viable cells in the treated sample and that in sham-treated samples [36].

### 2.10. Fast Halo Assay (FHA)

The assay was carried out as previously described [38]. Briefly, after the treatments, the cells were resuspended in ice-cold PBS/5 mM EDTA: 25 μL of this suspension was diluted 1:1 with 2% low melting agarose and sandwiched between a fully frosted agarose-coated slide and a coverslip. After complete gelling, the coverslips were removed and the slides immersed in NaOH 300 mM for 15 min. Ethidium bromide (10 μg/mL) was directly added to NaOH during the last 5 min of incubation. The ethidium bromide-labelled DNA was photographed with a Leica DMLB/DFC300F fluorescence microscope (Leica Microsystems, Wetzlar, Germany) and the micrographs processed with Scion Image software (Scion Corporation, Frederick, MD, USA). The number of DNA fragments diffusing out of the nuclear cage was determined by calculating the nuclear diffusion factor, which is the ratio between the total area of the halo plus the nucleus and that of the nucleus. The data are expressed as the relative nuclear diffusion factor, calculated by subtracting the nuclear diffusion factor of the control cells from those of the treated cells. 

### 2.11. Expression of Inflammatory Genes: RNA Isolation cDNA Synthesis and Quantitative Real-Time PCR

The total RNA from the treated cells was extracted according to the E.Z.N.A. total RNA kit manual (VWR International, Milan, Italy). An E.Z.N.A RNase-Free DNase I set (VWR International, Milan, Italy) digestion step was performed on all of the RNA samples before subsequent reactions.

The quantity of RNA was estimated spectrophotometrically at 260 nm (SpectraMax QuickDrop Micro-Volume Spectrophotometer, Molecular Devices, San Jose, CA, USA).

Reverse transcription of cDNA was performed from 500 ng of total RNA using the PrimeScript™ RT Reagent Kit (Takara Bio Europe, Saint-Germain-en-Laye, France).

From the mRNA expression analyses, the cDNA products were subjected to real-time SYBR-green-based quantitative PCR on a StepOnePlus™ Real-Time PCR System (Applied Biosystems, Monza, Italy) in a final volume of 20 ul using a Power Up master mix (Life Technologies) and 0.3 uM of the primer pairs reported in Table 1.

The primers used for the PCR experiments were previously designed and used by Calcabrini et al., 2017 and Coppari et al., 2021 [39,40].

The relative levels of target mRNA expression were normalized to values obtained for the tata-binding protein (TBP), used as a “housekeeping gene” simultaneously with the experimental samples.

The RT-PCR conditions were: 50 °C for 2 min, 95 °C for 2 min followed by 40 cycles of two steps—95 °C for 15 s and 60 °C for 60 s and, finally, three steps—95 °C for 15 s, 60 °C for 60 s and 95 °C for 15 s.

For miRNA analysis, human miR-146a was quantified by RT–qPCR using TaqMan MicroRNA assay (Applied Biosystems, Foster City, CA, USA) according to the manufacturer’s guidelines. The RT–qPCR data were standardized to RNU44 (reference miRNA).

The product specificity was examined using dissociation curve analysis. The results were calculated using the delta–delta Ct method (2^− ΔΔCt^) and were expressed as fold change related to untreated control (CTRL).

Each sample was tested in triplicate by RT–qPCR.

### 2.12. Enzyme-Linked Immunosorbent Assay (ELISA)

The levels of secreted IL-6 in the cell culture supernatant were determined using a human IL-6 Quantikine ELISA Kit according to the manufacturer’s instructions.

Supernatant samples were collected after 24 h recovery time and tested using IL-6 (DY206, R&D Systems, Minneapolis, MN, USA).

### 2.13. In Vitro Wound-Healing Assay

The HC-a were seeded in a six-well TC plate, incubated at 37 °C and 5% CO_2_ and allowed to grow to confluence as monolayers. The monolayers were subjected to a mechanical scratch wound, horizontal along the flask, using the tip of a sterile pipette. Subsequently, the medium of each flask was removed, the cells washed with PBS solution and grown for 24 h with fresh medium containing APEX, FR, ST or EFR. The untreated cells were used as a control. Micrographs of the lesion areas were taken either immediately after scratching (t_0_) or after 24 h using a phase-contrast microscope (Olympus I ×51 10× objective). To evaluate the wound closure, the images were analyzed with the ImageJ software 1.52a to select the total area of the wound region. The percentage of wound closure was calculated using the following equation [40].

[(wound area t_0_ − wound area t_24_)/wound area t_0_] × 100 (1)

### 2.14. Statistical Analysis

The statistical analyses were performed using GraphPad Prism version 8.02 for Windows (GraphPad Software version 8.02.263).

The variables among the treated samples of FHA were compared using one-way ANOVA with Dunnett’s post hoc. The differences between the samples were considered significant if the *p*-values were <0.05.

The two-tailed paired Student’s *t*-test was used for the miR-146a, IRAK-1 and IL-6 analyses. The results were considered significant at the level of *p* < 0.05. All of the experiments were conducted in triplicate. Linear regression analysis was used to calculate the EC_50_ values from the DPPH test and Fe^2+^-chelating ability assay.

The level of the expression gene measured by qRT–PCR and the IL-6 protein level were analyzed using a one-way ANOVA.

## 3. Results

### 3.1. Extract Characterization

The main biologically-relevant compounds identified were the phytosterols ST, campesterol, stigmasterol and the triterpenes EFR and FR (Table 2), as previously described by Ferrini et al. [6]. This extract, obtained from the same plants cultivated in [6], was used for all experiments.

### 3.2. Cytotoxicity and Genotoxicity of APEX

**Figure 1:**
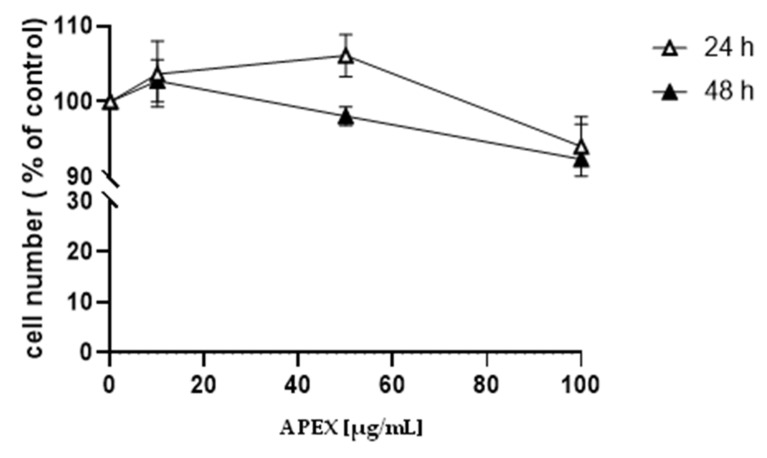
Effect of APEX on U937 cell growth. U937 cells were treated with increasing concentrations of APEX for 24 h and grown for a further 24 and 48 h in APEX-free medium. The number of viable cells was then determined using the trypan blue exclusion assay. Values represent the means ± SEM (standard error of the mean) of at least three independent determinations.

The effect caused by the continuous exposure to increasing concentrations of APEX on the growth of human promonocytoid U937 cells is shown in Figure 1. Concentrations up to 100 µg/mL did not significantly affect cell growth. 

**Figure 2:**
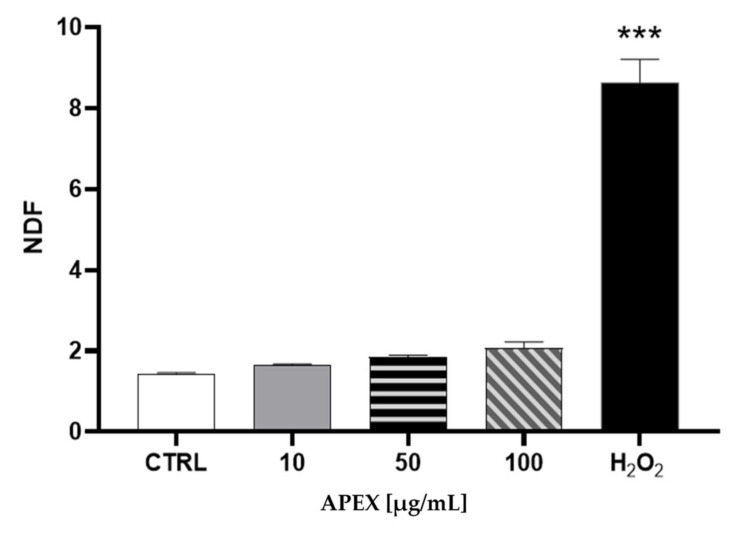
Effect of APEX on U937 nuclear DNA. Cells were treated as detailed in Figure 1 and then assayed for DNA damage. The extent of DNA breaks is expressed as NDF (see the Methods section). The effect of H_2_O_2_ (100 µM for 2 h) included as a positive control is also shown. One-way ANOVA with Dunnett’s post hoc test. *** *p* < 0.0001 CTRL vs. H_2_O_2_ 100 µM.

The analysis of nuclear DNA in cells exposed to the same range of APEX concentrations shows no significant DNA strand scission (Figure 2).

### 3.3. Antioxidant Activity

The antioxidant capacity of APEX—and of its three main components of FR, ST and EFR—was investigated using two different acellular tests, namely, the DPPH and the Fe^2+^-chelating ability assays. Concentrations ranging from 10 µg/mL to 1000 µg/mL of APEX showed a remarkable, dose-dependent activity in both DPPH free-radical scavenging effect and Fe^2+^-chelating capacity. 

**Figure 3:**
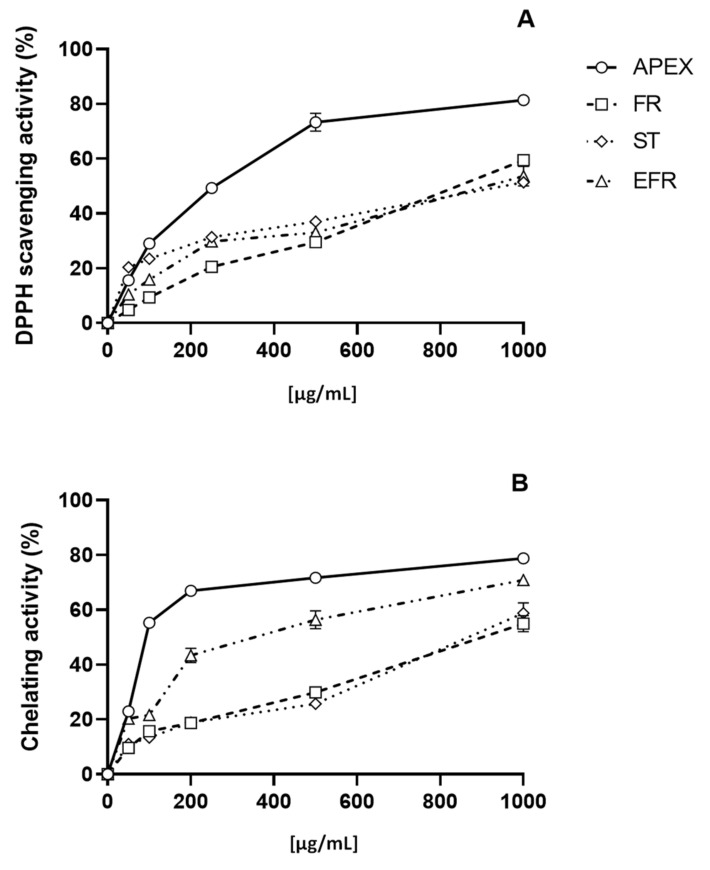
In vitro antioxidant capacity. Scavenging effect as assayed with the DPPH test (**A**); chelating ability on ferrous ions (**B**). Each value is the mean ± SEM of three independent measurements.

Similarly, the three bioactive components of APEX tested separately were found to exert dose-related scavenging and chelating activities, although to a lesser extent compared to the whole extract (Figure 3).

The EC_50_ values for APEX, FR, ST and EFR calculated from the results of the DPPH and Fe^2+^-chelation assays are reported in Table 3.

**Figure 4:**
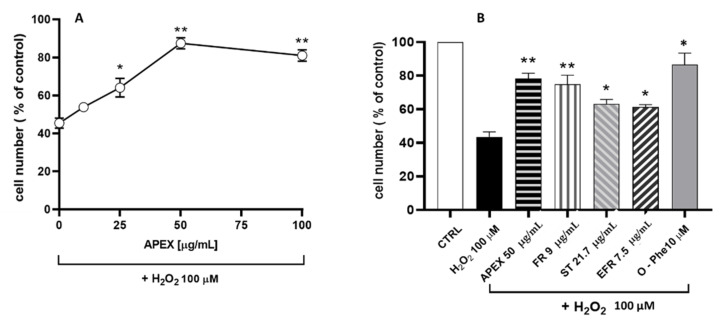
(**A**) Effect of APEX, FR, EFR or ST preincubation on the H_2_O_2_ cytotoxicity in U937 cells. (**A**) Cells were preincubated for 24 h with increasing concentrations of APEX or (**B**) with APEX (50 µg/mL), FR, EFR or ST (given at the same amounts contained in 50 µg/mL of APEX) and then treated with 100 µM H_2_O_2_ for 1 h. The viability was determined after 24 h growth in fresh, complete culture medium. Also shown is the effect of 30 min pretreatment with 10 µM of the iron-chelator *o*-phenanthroline (O-Phe) included as a positive control. Each point represents the mean ± SEM from four separate determinations, each of which was performed in duplicate. * *p* < 0.05 and ** *p* < 0.005 (one-way ANOVA) compared to control cells.

The second set of experiments aimed to evaluate the antioxidant capacity of APEX in cultured-cell systems. To this end, the increasing concentration assessment of APEX on the cytotoxic response of U937 promonocytoid cells exposed to a bolus of H_2_O_2_ was investigated. The concentrations tested were capable of mitigating oxidative challenge cytotoxicity (Figure 4) in a dose-related fashion.

In parallel, the active compounds present in APEX were separately tested under the same conditions; for comparative purposes, their concentrations were the same, contained in 50 µg/mL APEX. The results obtained (Figure 4) also indicate that the three compounds tested separately behaved similarly to APEX, although none of them was more active than the whole extract.

**Figure 5:**
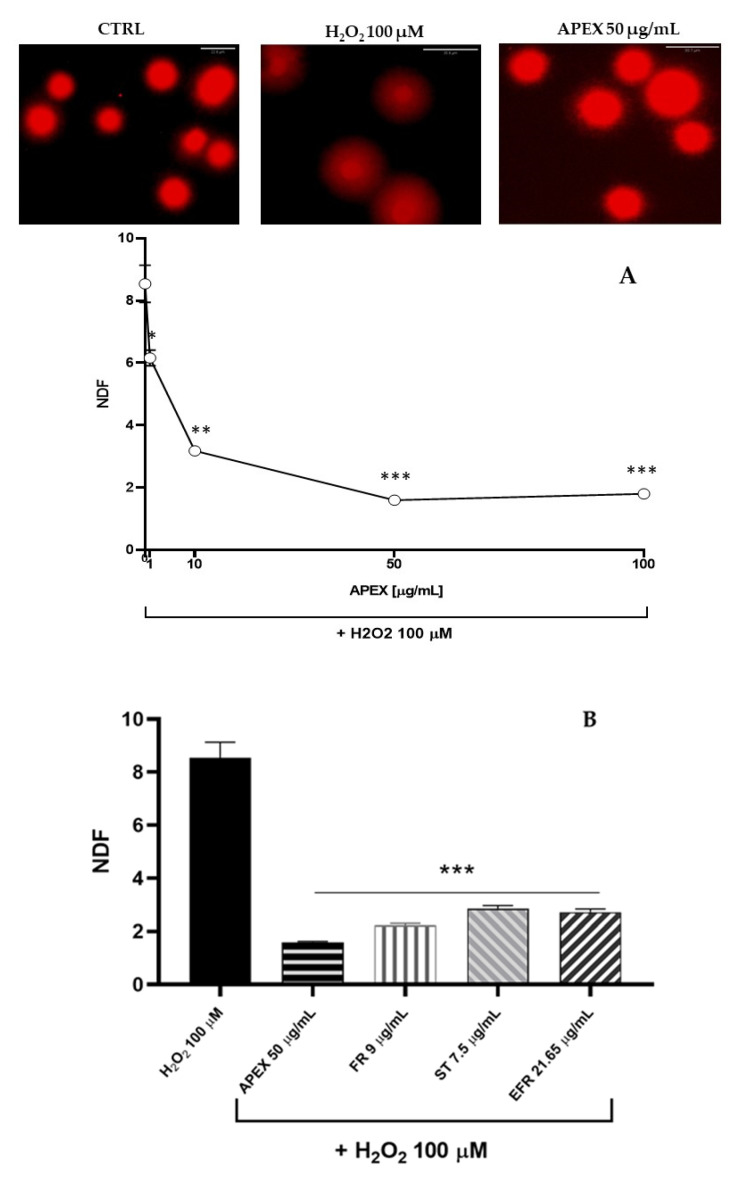
Effect of APEX and its major components on oxidant-induced DNA breaks. (**A**) Representative micrographs of U937 cells exposed to H_2_O_2_ preincubated for 24 h in the absence or presence of increasing concentrations of APEX; (**B**) extent of DNA lesions in the nuclear DNA from cells preincubated for 24 with APEX, FR, ST or EFR prior to oxidative challenge. Values are expressed as NDF and are the means ± SEM of three independent experiments. * *p* < 0.05, ** *p* < 0.001 and *** *p* < 0.0001 *vs.* H_2_O_2_.

Next, we investigated the ability of APEX, FR, ST and EFR to prevent the oxidant-induced DNA damage in U937 cells (Figure 5) [41].

As expected, H_2_O_2_ challenge (1 h, 100 µM) caused a significant increase in the halo size (see the Methods section), which reflects the high accumulation of DNA single-strand breaks.

Interestingly, preincubation with APEX at three different concentrations dose-dependently prevented DNA strand scission (Figure 5). Again, the three bioactive compounds tested separately were capable of affording genoprotection to an extent similar to APEX (FR and EFR) or slightly, but significantly, lower (ST).

### 3.4. Anti-Inflammatory Activity

The ability of APEX, as well as that of its three main components, to counteract the in vitro inflammatory effects promoted by lipopolysaccharide (LPS) was evaluated.

**Figure 6:**
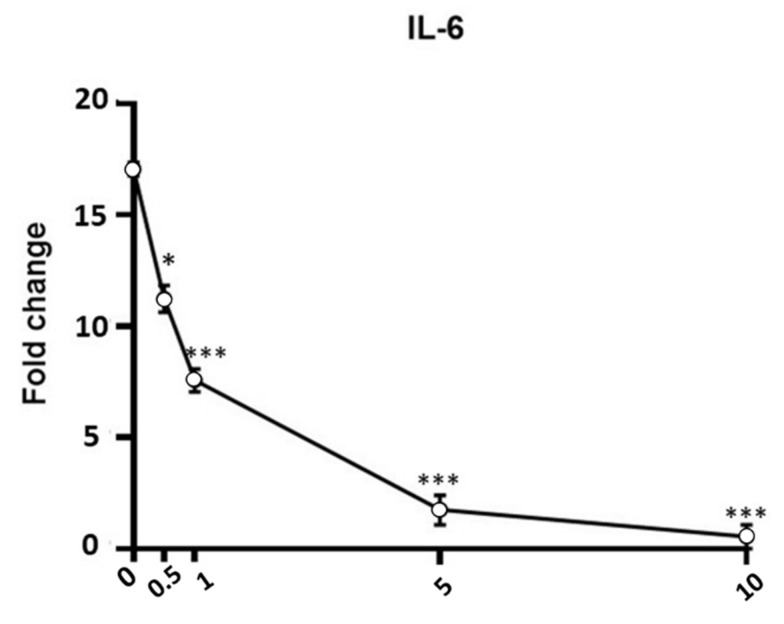
Effect of APEX on IL-6 gene expression in LPS-stimulated cells. U937 cells were preincubated (24 h) with increasing concentrations of APEX and then grown for a further 12 h with LPS (1 µg/mL); the expression level was determined immediately after LPS treatments. Data are expressed as fold increase compared to untreated control cells, and are the means ± SEM of at least three independent experiments. * *p* < 0.05, *** *p* < 0.0001, compared to LPS-stimulated cells without APEX (one-way ANOVA).

**Figure 7:**
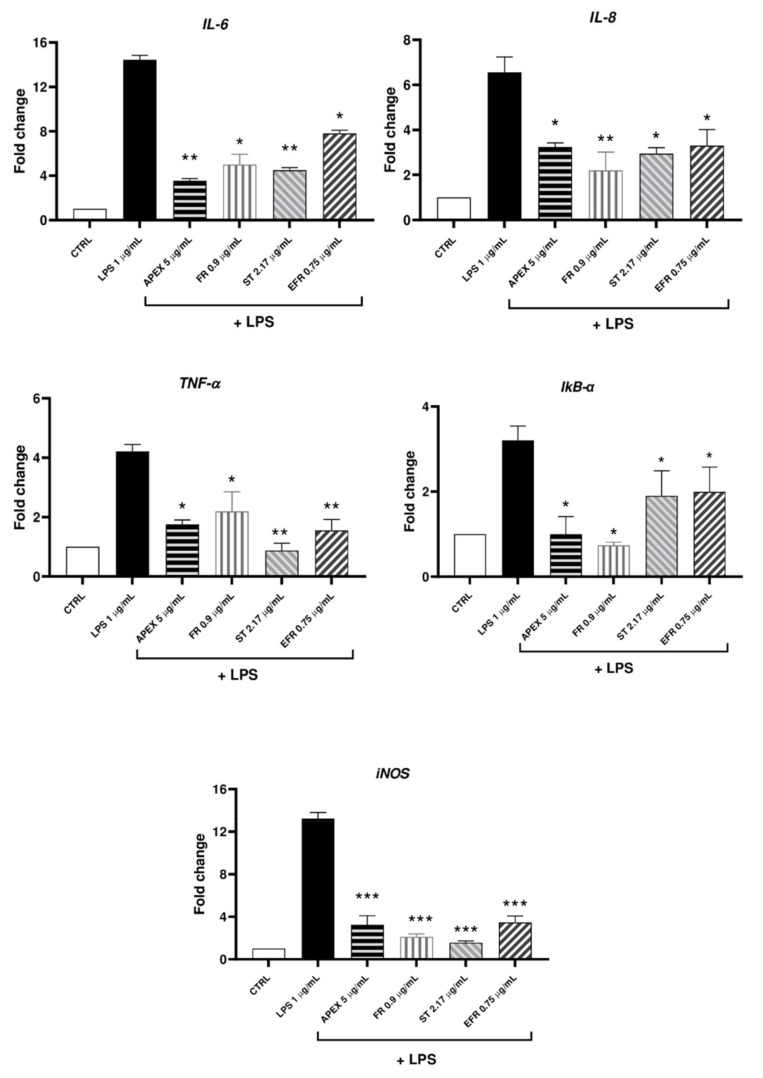
mRNA levels of cytokines and selected inflammation markers (*IL-6, IL-8, TNF-a, IkB-a, iNOS*) in LPS-stimulated U937 cells. Cells were preincubated (24 h) with APEX, FR, ST and EFR and then grown for a further 12 h with LPS (1 µg/mL); the expression level was determined immediately after treatments. Data are reported as fold increase compared to untreated control cells. Data are the means ± SEM of at least three independent experiments. * *p* < 0.05, *p* ** < 0.001, *p* *** < 0.0001, compared to LPS-stimulated cells (one-way ANOVA).

In the first set of experiments, we analyzed the gene expression levels of inflammatory markers (*IL-6*, *IL-8*, *TNF-α*, *IkB-α*, *iNOS*) after exposure to LPS (1 µg/mL for 12 h) in the absence or presence of APEX or of its separate components. As shown in Figure 6 and Figure 7, an increased expression of all of the selected genes could be observed in LPS-treated cells. Among the concentrations tested, 5 µg/mL APEX exerted a nearly-maximal effect on IL-6 expression (Figure 6) as well as on the other cytokines (not shown); hence, 5 µg/mL APEX and the corresponding amounts of FR, EFR and ST (when tested alone) were used in the next sets of experiments. Importantly, pretreatment with APEX, FR, ST and EFR significantly prevented LPS-induced overexpression of all of the selected genes.

In order to see whether the LPS-induced increased gene expression resulted in a parallel increase in protein accumulation, the amount of IL-6 released from U937 cells in the growth medium 24 h after the treatment was determined.

**Figure 8:**
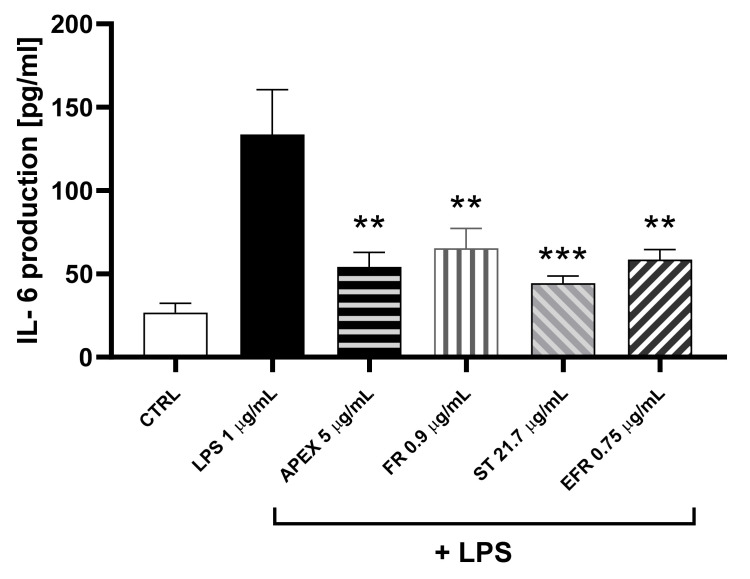
Effect of APEX and its major components on IL-6 protein levels. U937 cells were preincubated for 24 h with APEX, FR, ST e EFR; at the end of the 24 h, 1 µg/mL LPS was added and cells incubated for a further 12 h. IL-6 levels were determined after 24 h growth in drug-free medium by ELISA assay. Results are expressed as µg/mL of medium. Values represent the means ± SEM (n = 3). One-way ANOVA was used to measure the significance of differences between groups. *** *p* < 0.001 LPS vs. APEX- and ST-treated cells or control vs. LPS vs. FR- and EFR-treated cells ** *p* < 0.01.

The results of the ELISA assay showed that the IL-6 protein levels reflected the gene expression observed in each experimental condition (Figure 8).

To dissect the biological mechanisms at the basis of the APEX effects reported above, we next measured the gene expression of the biomarker combination of *miR-146a* and its target protein IL-1 receptor-associated kinase (IRAK-1) involved in the regulation of the IL-6 activation pathway, where IRAK-1—which actively participates in the Toll-like receptors signaling—is regulated in a negative feedback fashion by *miR-146a* expression [42]. Therefore, we evaluated the regulation of *miR-146a* and *IRAK-1* in APEX-preincubated cells.

**Figure 9:**
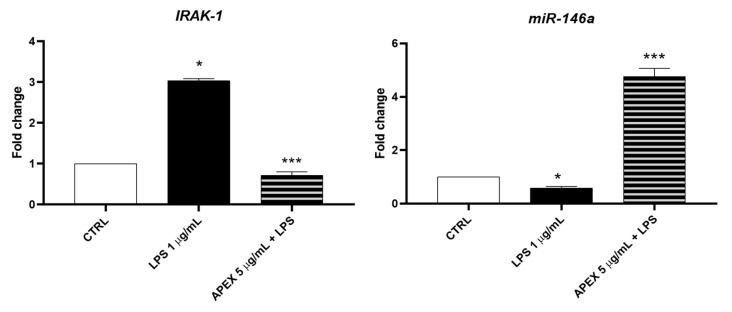
Effect of APEX on *IRAK-1* and *miR-146a* expression levels in LPS-stimulated U937 cells. Treatments were the same as in Figure 6. Results are reported as fold change compared to control. Results are the mean ± SEM of three experiments. One-way ANOVA was used: * *p* < 0.05 CTRL vs. LPS; *** *p* < 0.01 LPS vs. APEX + LPS.

We found that *IRAK-1* and *miR-146a* were significantly increased or reduced, respectively, after 12 h of treatment with LPS (1 µg/mL). Interestingly, APEX promoted an opposite response on LPS-stimulated cells in that it increased the expression of miR-146a, while decreasing that of IRAK-1 (Figure 9).

### 3.5. Wound-Healing Repair Activity

The correlation between APEX’s antioxidant/anti-inflammatory effects and its potential ability to heal wounds was examined using the monolayers’ scratch assay.

**Figure 10:**
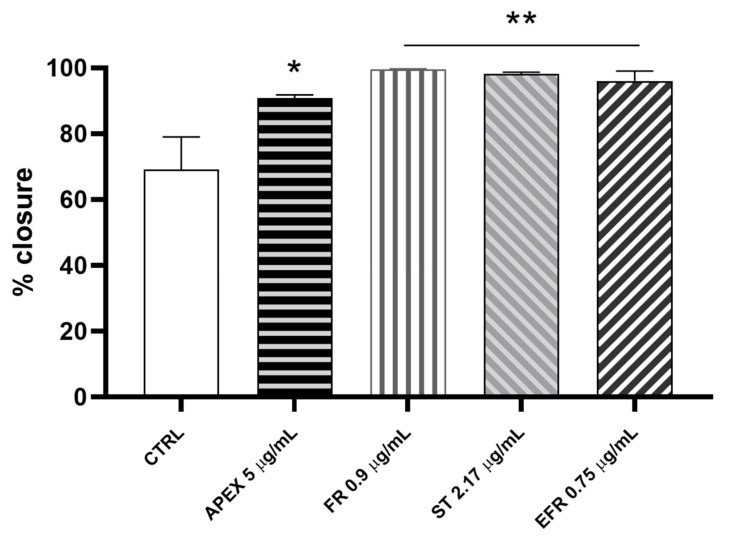
Wound-healing after APEX, FR, ST and EFR treatment. The graph shows the percentage of wound closure compared to the untreated HC-a cells after 24 h. The values represent the mean ± SEM of three independent experiments. * *p* < 0.05, ** *p* < 0.001 vs. CTRL (one-way ANOVA).

We investigated the wound-healing properties of APEX and its individual constituents on HC-a human chondrocytes (Figure 10).

We found that the percentage of wound closure after 24 h of APEX supplementation improved significantly compared to control cells (91% vs. 61%, respectively). Even better results were afforded by FR (99.7%), ST (98.3%) and EFR (96%) treatment (Figure 10).

## 4. Discussion

A variety of products based on the bioactivities of plant extracts or purified compounds are increasingly being developed and globally consumed [43,44], with an estimated value of 140 billion dollars by 2024. Indeed, a wide variety of plants and botanicals are capable of affording health benefits, mainly involving the prevention and/or the adjunctive treatment of some chronic diseases [6]. When extracted from edible plants, these compounds are termed “nutraceuticals”.

In this context, the present research was undertaken to characterize two relevant biological—namely, anti-inflammatory and antioxidant—activities of the ethanolic extract of *C. sativa* roots grown in an aeroponic setting.

Aeroponics was chosen because it allows the collection of clean roots free from the contaminants and parasites that can be found in soil-cultivated plants [6]; it yields higher amounts of raw material and bioactive constituents; and it meets the standards of organic cultivation, which today represents a commercially valuable and desirable end point. Moreover, the aeroponic system is industrially scalable in a cost-effective manner. The composition of APEX (Table 1) was previously characterized and compared with the ethanolic extract obtained from soil-cultivated plants in a recent study [6] by our group, where it was also shown that the per-plant yield of roots’ bioactives was higher in aeroponics than in soil-cultivated plants.

The present work demonstrates that APEX and its components, tested at non-toxic concentrations, act as antioxidants in either cell-free or in cell-based determinations.

In acellular systems, the antioxidant activity of the extract in toto was higher compared to the single components tested at concentrations equivalent to those present in the whole APEX. This suggests that these molecules do not interact negatively each other, but rather produce an additive-like effect. APEX and its major constituents were active in both DPPH and Fe^2+^-chelation assay, indicating that they act in a bimodal pattern, i.e., through radical scavenging (direct DPPH quenching) and transition metal chelation (formation of Fe^2+^ chelates), a mixed mode of action that is rather common in plant-derived bioactive molecules [45].

When tested in cell systems, APEX, FR, EFR and ST retained their antioxidant activity against H_2_O_2_-induced cytotoxicity and genotoxicity toward U937 cells. This finding lends further support to the above notion that both radical scavenging and iron-chelating capacities contribute to the whole antioxidant activity [45]. However, since it is known that only iron chelators, unlike radical scavengers, are capable of mitigating nuclear DNA oxidative lesions [45], it is plausible that the chelating capacity might have a prominent role in APEX’s and its constituents’ antioxidant activity.

When tested separately, the antioxidant activity of FR, EFR and ST in cell systems was comparable to that of APEX; however, they did not seem to exert any additive-like effect, as observed in the cell-free tests. The loss of additive interactions is likely dependent on the fact that—differently from the acellular settings—the relative activity of the compounds given alone or in the phytocomplex may vary significantly, as other components in APEX may, for example, compete with or affect their cellular pharmacokinetics and pharmacodynamics.

The anti-inflammatory activities of some medicinal plants have been attributed to their content of terpenes, such as FR, that shows a potent in vivo anti-inflammatory activity [23]. Importantly, as recently discussed by Ryz et al. [46], the anti-inflammatory properties of the roots of *C. sativa* have a solid ethnobotanical history and would deserve much more attention. Although there is currently no research available about their relative contribution, FR and EFR are likely candidates accounting for *C. sativa* roots’ anti-inflammatory activity. Hence, in addition to whole APEX, we addressed our experimental approach on the two triterpenoids and, considering its quantitative prevalence, also on ST.

Here, we show that the LPS-stimulated gene expression of relevant inflammation markers, namely, *IL-6, IL-8, TNF-a, IkB-α* and *iNOS*, was efficiently prevented by APEX, and that FR, EFR and ST given alone were also effective to a similar extent.

The relevant anti-inflammatory capacity was further strengthened by the finding that APEX and its major components were all capable of preventing the synthesis and the extracellular release of IL-6.

Given the importance of IL-6 in inflammation and its recently highlighted implications in COVID-19 pathogenesis [47,48] we investigated the mechanistic interactions of APEX and its selected constituents with the IL-6 gene expression pathway. In particular, we focused on the expression of *miR-146a* and *IRAK-1*. IRAK-1 is involved in downstream TLR4 (Toll-like receptor-4) signaling pathway activation; TLR4 represents a key receptor on which different stimuli converge to induce a proinflammatory response. In this condition, TLR4, through the IRAK-1 mediator, leads to the activation of the NF-κB transcriptional complex, and finally the production of proinflammatory cytokines, including IL-6. Conversely, miR-146a inhibits the transcriptional complex NF-κB activation and the ensuing proinflammatory cytokine production by directly targeting IRAK1 expression [42,49]. According to this notion, we found that upon stimulation of U937 cells with LPS, IRAK-1 was overexpressed, while miR-146a was underexpressed; more interestingly, we also found that APEX promotes miR-146a overexpression under the same LPS-stimulus conditions. Hence, it is plausible that the miR-146a-a overexpression may be causally linked to the parallel downregulation of IRAK-1 and mechanistically involved in the overall anti-inflammatory activity of APEX and its major components reported herein.

The fact that other natural products displaying anti-inflammatory capacity have been reported to induce the upregulation of miR-146a [50] lends further support to the above inference.

Hence, APEX shows remarkable antioxidant and anti-inflammatory activities in cultured cells systems. From a quantitative point of view, APEX produces its maximal anti-inflammatory capacity at a concentration 10-fold lower than the maximal antioxidant activity. This finding suggests that APEX and its major components might be preferably exploited for anti-inflammatory purposes. In particular, although this is mere speculation, the effect of APEX on IL-6 levels via the miR-146a/IRAK-1 pathway might be pharmacologically relevant, since it could cooperate with the drugs directly targeting IL-6, such as anti-IL-6 receptor mAbs (e.g., sarilumab, tocilizumab) and anti-IL-6 mAbs [51], which act at a separate, downstream level.

One of the problems affecting the nutraceutical/pharmacological exploitation of phytocomplexes is their scarce absorption and bioavailability through the oral route. In fact, no data are available for FR and EFR absorption. However, absorption is not always a limiting factor in treatments including, but not restricted to, topical use for dermatological conditions and the medication of wounds [52]. Indeed, chronic inflammatory bowel diseases (CIBD) [53] can also be treated with drugs exhibiting scarce or null absorption through the gut, as they can act in a topical-like fashion. In this light—bearing in mind the lack of information on its pharmacokinetic profile—APEX would be an attractive candidate for the adjunctive management of these severe diseases. Furthermore, IL-6 and TNF-α, which are significantly downregulated and inhibited by APEX, are among the major pathogenetic actors of CIBD [54]; IRAK-1, targeted by APEX, has been found to be involved in ulcerative colitis and Crohn’s disease, and it is increasingly expressed during the active phases of these conditions [55]. Incidentally, FR has recently and independently been reported to ameliorate chemically-induced colitis in mice through anti-inflammatory mechanisms involving decreased proinflammatory cytokines IL-1β and IL-6 accumulation, and the inhibition of autophagy via interaction with the AMPK-mTOR signaling pathway [56].

Another clinical setting where pharmacokinetic limitations are circumvented is the infiltrative treatment of arthrosic conditions, in which the drugs can be directly delivered within the articular space [57]. In osteoarthrosis, chondrocytes represent a key cellular target of the degradative process. Here, we show that HC-a human chondrocytes undergo an accelerated wound-closure process in the presence of APEX and its components. Reportedly, in the initial phase, the wound-closure process leads to an immediate acute inflammation involving different cytokines and growth factors, with IL-6 playing a prominent role [58]. Furthermore, at later stages, a crucial step to achieve complete wound healing is represented by IL-6 downregulation. Chondrocytes wound closure after monolayers’ scratching has been used as a model to mimic chondrocytes’ outgrowth necessary in cartilage repair [59]. Notably, when functionally benchmarked in the experimental wound-healing assay, APEX and its major constituents significantly promoted/accelerated the wound closure process—an effect that is likely related to the anti-inflammatory and antioxidant properties of APEX, FR, EFR and ST. Along the same lines, it is important to mention that the efficiency of gut mucosa wound closure is known to contribute to the amelioration of the CIBD conditions [60] discussed above.

Another important feature of *C. sativa* roots and of APEX, not secondary to the above considerations, is the very high content of ST [6], whose potential in the adjunctive management of hyperlipidemia and prostate hypertrophy has been fully recognized. In keeping with our data, ST has also been shown to exert remarkable anti-inflammatory effects; indeed, Liao et al. [61] reported that ST suppressed the secretion of inflammatory factors from keratinocytes and macrophages induced by peptidoglycan, and significantly reduced the expression of NLRP3, a key component of NLRP3 inflammasomes, and inhibited the activation of caspase-1 and of NF-κB.

Aeroponic roots and APEX may then represent an excellent source of this sterol, far better than the popular *Serenoa repens* extract, which contains an amount of ST one hundred times lower [6].

For the sake of completeness, it is important to consider that the major limitation of this study is represented by the fact that no in vivo approach has been included; consequently, the perspectives raised in our study are speculative. However, the high anti-inflammatory potency and the fair antioxidant capacity of APEX, along with the rational mechanistic ground proposed herein, are promising with regard to the exploitation of APEX in the above widespread and socially relevant conditions.

## 5. Conclusions

In this study, we evaluated the biological activity of *C. sativa* root ethanolic extract in toto and of its major constituents, and found that they possess remarkable anti-inflammatory and antioxidant activities. These properties could be exploited in a wide range of applications in health promotion, including pharmaceutical, nutraceutical, dermatological and food products obtained through the economically advantageous, reproducible and sustainable aeroponic cultivation system.

## Figures and Tables

**Table 1:** Primer sequences for real-time PCR.

Gene	Primer	Sequence	Accession Number
*IL-6*	Forward	AGGGCTCTTCGGCAAATGTA	NG_011640.1
*IL-6*	Reverse	GAAGGAATGCCCATTAACAACAA	NG_011640.1
*IRAK-1*	Forward	CAGACAGGGAAGGGAAACATTTT	NG_008387.1
*IRAK-1*	Reverse	CATGAAACCTGACTTGCTTCTGAA	NG_008387.1
*TNF- α*	Forward	CTGCTGCACTTTGGAGTGAT	NG_007462.1
*TNF-α*	Reverse	TCTCAGCTCCACGCCATT	NG_007462.1
*IL-8*	Forward	GCAGAGGGTTGTGGAGAAGT	NG_029889.1
*IL-8*	Reverse	GCTTGAAGTTTCACTGGCATC	NG_029889.1
*IkB-α*	Forward	GGTGCTGATGTCAATGCTCA	NG_007571.1
*IkB-α*	Reverse	ACACCAGGTCAGGATTTTGC	NG_007571.1
*iNOS*	Forward	CCCTTCAATGGCTGGTACATGG	NG_011470.1
*iNOS*	Reverse	ATGTTGATCTCAACGACAGCC	NG_011470.1
*TBP*	Forward	TGCACAGGAGCCAAGAGTGA	NG_008165.1
*TBP*	Reverse	CACATCACAGCTCCCCACCA	NG_008165.1

Note: *IL-6*, interleukin-6; *IL-8*, interleukin-8; *IRAK-1*, interleukin-1 receptor-associated kinase 1; *IkB-α*, nuclear factor of kappa light polypeptide gene enhancer in B-cells inhibitor alpha; *TNF-α*, tumor necrosis factor-alpha; *iNOS*, inducible nitric oxide synthase; *COX-2*, cyclooxygenase-2; *TBP*, tata-binding protein.

**Table 2:** Content of the main bioactive compounds present in aeroponic *C. Sativa* roots.

Compound	Amount (µg/100 mg Dry Roots) ^a^
Campesterol	17.2 ± 0.4
Stigmasterol	20.2 ± 0.2
β-Sitosterol	68.7 ± 1.1
Epi-Friedelanol	23.9 ± 0.1
Friedelin	28.8 ± 1.2
Total	158.8 ± 1.9

^a^ Data are expressed as the mean value ± standard deviation; n = 3 repetitions.

**Table 3:** EC_50_ DPPH and Fe^2+^-chelating activity.

Antioxidant Activity (EC_50_ Values, μg/mL)				
	APEX	Friedelin	β-Sitosterol	Epi-Friedelanol
DPPH scavenging activity ^a^	420.1 ± 2.1	832.4 ± 1.7	920.2 ± 3.4	875.1 ± 1.7
Chelating activity ^a^	385.5 ± 3.0	883.5 ± 7.2	858.8 ± 6.2	547.6 ± 6.3

(^a^) Each value represents the mean ± SEM of three independent measurements. EC_50_ values calculated from linear regression analysis of the curves depicted in Figure 3.

## Data Availability

All of the data is contained within the article.

## Abbreviations

APEX	Ethanolic extracts of *C. sativa* aeroponic roots
CIBD	Chronic inflammatory bowel diseases
EFR	Epi-friedelanol
FR	Friedelin
SEM	Standard error of the mean
ST	β-Sitosterol
